# 肺高分化胎儿型腺癌1例报告并14例文献复习

**DOI:** 10.3779/j.issn.1009-3419.2010.08.18

**Published:** 2010-08-20

**Authors:** 同梅 张, 娜 秦, 宝兰 李

**Affiliations:** 101149 北京，北京胸科医院综合科 General Department, Beijing Chest Hospital, Beijing 101149, China

肺高分化胎儿型腺癌（well- dif ferentiated fetal adenocarcinoma of lung, WDFA）是一种罕见的肺的低度恶性肿瘤，文献^[1]^报告其发病率为所有肺部肿瘤的0.25%-0.5%。我们报告1例肺高分化胎儿型腺癌，结合文献复习探讨其临床特征、流行病学资料、治疗及预后，提高医务人员对WDFA的认识。

## 1 临床资料

患者，男性，26岁。主诉“发现肺内阴影两年，咳嗽、胸闷、盗汗20天”入院。两年前体检发现右上肺阴影，无咳嗽、咳痰、发热等症状，当地医院按肺结核给予四联正规抗结核治疗1年余，治疗期间多次复查胸片右上肺病灶无明显变化，后遵医嘱停药。此次20天前因咳嗽、胸闷、盗汗就诊当地医院，胸片发现右上肺阴影增大（图 1），胸CT（图 2）示右上尖后段软组织团块影，纵隔淋巴结不大。为进一步诊治入北京胸科医院。患者既往无烟酒不良生活嗜好，无肿瘤相关疾病家族史。体格检查：未见明显阳性体征。PPD：硬结20 mm ×23 mm，ESR 2 mm/h，血肿瘤标志物癌胚抗原、鳞癌抗原、胃泌素蛋白前体、神经烯醇化酶、角质蛋白均阴性。血、尿、便常规、肝肾功能及电解质均正常。头部MRI、骨扫描、腹部彩超、淋巴结B超均未见异常，纤支镜未见明显异常，入院诊断考虑肺癌可能性大。入院后行CT定位下肺穿刺活检，病理结果为：肺组织及支气管粘膜组织慢性炎症，未见肿瘤细胞。外科开胸探查，术中见肿瘤位于右上叶后段，约7 cm×5 cm×5 cm，质地硬韧，表面凹凸不平，冰冻病理考虑为癌瘤组织，行右肺上叶切除术，加纵隔淋巴结清扫术。术后病理为：肺高分化胎儿型腺癌（PT2N0M0）（图 3）。

**1:**
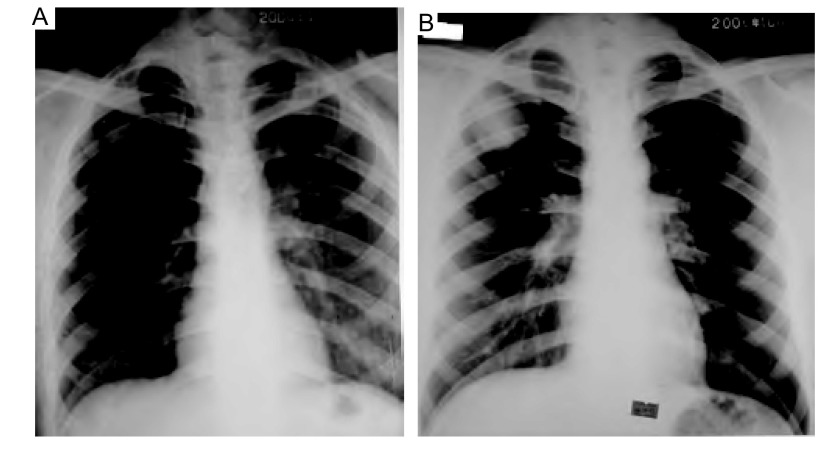
不同时期的胸片。A图显示常规体检时发现的右上叶片状影，B图示抗结核治疗后右上叶的团块影较前增大。 Chest X-ray of different period. A showing a patch lie in right upper lung when routine examing, and B showing a mass in the same place is larger than befor after anti-tuberculosis theraping.

**2:**
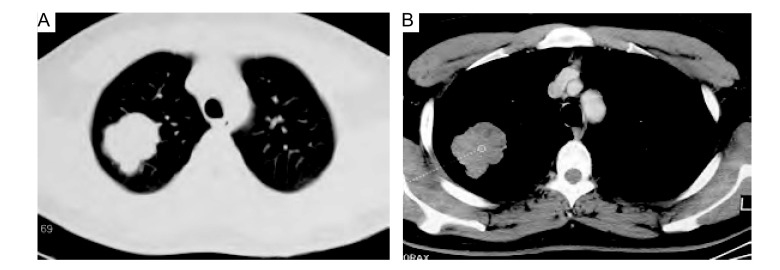
胸部CT。A肺窗和B纵膈窗示右上叶软组织团块影。 Chest CT. A pulmonary and B mediastinum windows CT showing a mass lies in right upper lung.

**3:**
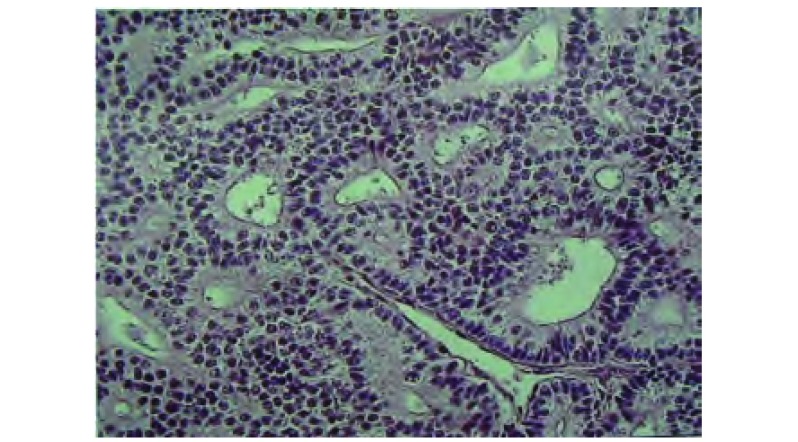
病理。癌细胞组成管状或腺样结构。 Pathology. Cancer cells are made of tubular or adenoid structure.

## 2 讨论

1981年WHO（世界卫生组织）分类和1995年AFIP（美军病理研究所）分类中，将WDFA划分为肺母细胞瘤，1999年第3版WHO和IASLC（国际肺癌研究协会）将WDFA列为腺癌的一个少见变异型，肺母细胞瘤归入“癌，伴多形性、肉瘤样、肉瘤成分”一组。2004年WHO肺和胸膜肿瘤组织学分类将WDFA归为腺癌的变异型。显微镜下癌细胞组成管状或腺样结构，无包膜，但与周围肺组织分界清楚。病理上高分化胎儿性腺癌的诊断要点：癌组织类似分化好的子宫内膜样癌，有核上和（或）核下糖原空泡；由实性上皮细胞巢构成的桑葚体；癌细胞核异型性不明显；有少量坏死和核分裂。1991年美国的Koss等^[1]^报告28例WDFA，平均年龄33岁，男13例，女15例，肿瘤大小1 cm-10 cm，平均4.5 cm，中位随访时间95个月，5年生存率为81%。2003年美国华盛顿沃特里德陆军医院的Difurio等^[2]^报告1例10岁男童病例，并复习文献7例10岁-19岁青少年WDFA病例。2006年美国的Paull等^[3]^报告1例47岁的女性患者并复习文献7例WDFA病例。2006年日本Sato等^[4]^报道1例36岁男性WDFA病例并复习日本国内25例病例。本文作者复习1991年-2007年国内外文献，沿用2004年WHO肺和胸膜肿瘤组织学分类，共搜集WDFA病例14例，结合本例分析汇总，如表 1。

**1:** 15例WDFA患者临床资料汇总表 Clinical data summary of 15 cases

Age	Gender	Symptom	Max diameter tumor (cm)	Location	Diagnosis pathway	Alive follow up (month)	Author	Nation country
9	Female	Chest pain and distress	4	L.U.L	Operation	?	Han JA^[5]^	Chinese
6	Male	Cough, sputum	5	L.U.L	Operation	17/alive	Kou YL^[6]^	Chinese
48	Female	Hemoptysis, chest distress	6	R.L.L	Operation	?	Guo AT^[7]^	Chinese
29	Female	Cough, suptum	1.2	R.M.L	Operation	2/alive	Tan MH^[8]^	Chinese
26	Male	No symptom	7	R.U.L	Operation	36/alive	This study	Chinese
29	Female	Chest pain, night sweat	3	L.U.L	Operation	18/alive	Sheehan K.M^[9]^	Ireland
47	Female	No symptom	3	L.U.L	Operation	?	Paull^[3]^	USA
41	Male	Intermittental cough	3	R.M.L	Operation	96/dead	Danilo N.^[10]^	Canada
49	Female	Sudden chest pain	6	R.M.L	Operation	9/alive	Politiek M J^[11]^	Holland
36	Female	Hemoptysis	4.4	L.U.L	Operation	?	Proctor L^[12]^	USA
10	Male	Cough, short breath	5.5	R.M.L	Operation	24/alive	Difurio M.J^[2]^	USA
48	Male	Chest pain, asthenia	3	L.U.L	Operation	22/alive	Shi HQ^[13]^	Chinese
27	Female	Cough, blood-steak	2.5	R.U.L	Operation	?	Fang GY^[14]^	Chinese
33	Female	No symptom	9	R.M.L	Operation	12/alive	Fujino S^[15]^	Japan
33	Female	No symptom	5	R.U.M.L	Operation	?	Okano T^[6]^	Japan

本文文献汇总男女发病无明显差异，发病年龄6岁-56岁，平均发病年龄33岁，以青壮年多见，发病年龄与既往文献报告一致。主要症状为咳嗽、咳痰、咯血、胸痛、胸闷等呼吸道症状为主，无特异性，故症状上不易与其它呼吸道疾病鉴别。影像学上双肺均可发，以左上肺和右中上肺多见，下叶少见，这与结核好发于双肺上叶相似，如果患者发病年龄较轻，又有相应的结核中毒症状，单凭胸片检查而不行CT等检查，往往容易误诊。该组病例肿瘤最大径范围1.2 cm-9 cm，平均4.5 cm，瘤体大小与既往文献报告一致。本病例行痰检、纤支镜及CT定位下肺活检均未能诊断，最后行手术确诊。结合本文文献病例均以手术确诊，故提示WDFA通过痰、纤支镜检和CT定位下肺穿刺活检等微创检查不易确诊，可能与本病病理分化较好，微创检查所取组织较少，不能提供有效的诊断标本等有关。本病是腺癌的一种变异性，低度恶性，有较长的生存期，以局部生长为主，不管肿瘤大小，很少发生远处转移和纵隔肺门淋巴结转移，手术切除率高。本文献复习中11例患者按照1998年国际抗癌联盟第6版肺癌TNM分期均为Ia-Ib期患者，均无肺门和纵隔淋巴结转移；其次本病对放化疗不敏感，结合本文文献大多数病例未行术后的辅助放疗和化疗，本文文献中6例患者无随访时间和存活状态的信息，其余患者在随访时间之内均存活，故提示早期根治手术可能是最佳的治疗手段，并有利于总生存的延长。故积极手术根治有助于早期诊断和治疗，减少误诊和误治。本病例术后已3年，未行放化疗，目前无任何症状，正常工作生活。

病理诊断上需与双向型肺母细胞瘤及胚胎肺型腺癌等鉴别，WDFA罕见，生长速度缓慢，易被误诊为良性肿瘤。本文结合既往文献资料提示WDFA平均发病年龄在30岁-40岁，男女无差别，儿童少见，以呼吸道症状为主，无特异的临床表现，双肺均可发，以上叶多见，很少发生肺门或纵隔淋巴结转移。微创检查不易确诊，绝大多数病例均通过手术确诊。为低度恶性肿瘤，有较好的预后，对放化疗不敏感，完全手术切除后的5年生存率能达到80%或以上^[1]^，早期诊断行手术切除是最主要的治疗方法。
